# Supplementary material for: Leveraging information between multiple population groups and traits improves fine-mapping resolution
Source: Nat Commun. 2023 Nov 10;14:7279. doi: 10.1038/s41467-023-43159-5 (PMC10638399; doi:10.1038/s41467-023-43159-5)
Supplement: Supplementary file 1 — Supplementary Information [file 41467_2023_43159_MOESM1_ESM.pdf]

Leveraging information between multiple groups and traits improves fine-mapping accuracy – Supplementary Information

F Zhou, O Soremekun, T Chikowore, S Fatumo, I Barroso, AP Morris, JL Asimit

Contents

1 Supplementary Figures 2

2 Supplementary Tables 7

3 Supplementary Methods 9

1 Supplementary Figures

Supplementary Figure 1: **Flashfm, MGflashfm and MGfm, are well-calibrated in EUR-EAS fine-mapping.** Coverage is measured as the probability that all causal variants are captured by the 99% credible set, estimated over 300 replications. Data are presented as the proportion of replications in which the 99% credible set contains all causal variants ± SEM, where SEM is the standard proportion error bound of a 95% confidence interval based on 300 observations. Flashfm-EUR and flashfm-EAS are multi-trait (single-group) fine-mapping for the indicated group and are well-calibrated in all settings, as are MGflashfm and MGfm. PAINTOR is not well-calibrated for all settings, while msCAVIAR is not well-calibrated for unequal sample sizes and for multiple causal variant settings. Within each panel the three simulation settings are shown as either having equal sample sizes of 10k each or sample sizes of 90k EUR and 10k EAS, and either two causal variants for each trait with one shared (trait 1: AD, trait 2: AC) or non-overlapping causal variants and one trait having a single causal variant (trait 1: AD, trait 2: C); any pair of causal variants have $r^2 < 0.5$.

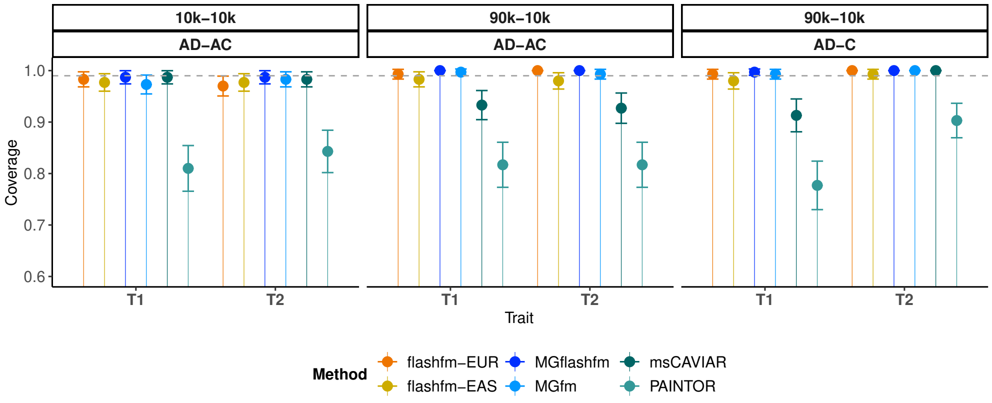

Supplementary Figure 2: **MGflashfm and MGfm attain the highest power amongst the methods.** Power is measured as the mean proportion of causal variants having PP above a certain threshold (e.g. 0.5 (left) or 0.9 (right)), estimated over 300 replications. The mean power is shown for each method, as indicated by the top of each bar; the distribution of the power estimates over the 300 replications is shown by violin plots, where width indicates frequency. There is a general pattern of highest power for MGflashfm and MGfm, and similarly high powers for the group-specific flashfm and PAINTOR. In all settings, there are two traits, each with two causal variants, of which one is shared (trait 1: AD, trait 2: AC); any pair of causal variants have $r^2 < 0.5$. The sample sizes are 90k EUR - 10k AFR, 90k EUR - 10k EAS, and 90k EUR - 40k EAS - 10k AFR.

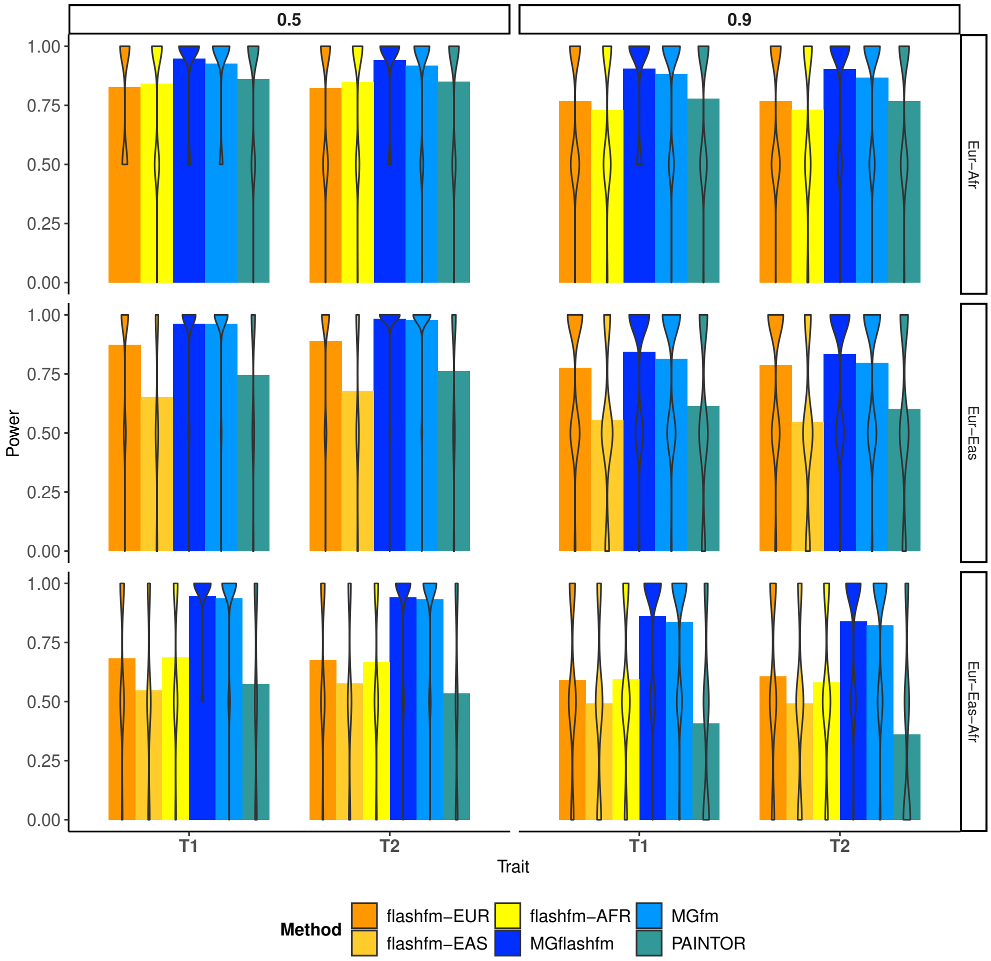

Supplementary Figure 3: **The flashfm methods have similarly low FDR.** FDR is measured as the mean proportion of non-causal variants having PP above a certain threshold (e.g. 0.5 (left) or 0.9 (right)). The mean FDR is shown for each method, as indicated by the top of each bar; the distribution of the FDR estimates over the 300 replications is shown by violin plots, where width indicates frequency. The FDR of MGflashfm, MGfm, and the group-specific flashfm are similarly low at PP threshold 0.9, but PAINTOR has very high FDR. In all settings, there are two traits, each with two causal variants, of which one is shared (trait 1: AD, trait 2: AC); any pair of causal variants have $r^2 < 0.5$. The sample sizes are 90k EUR - 10k AFR, 90k EUR - 10k EAS, and 90k EUR - 40k EAS - 10k AFR.

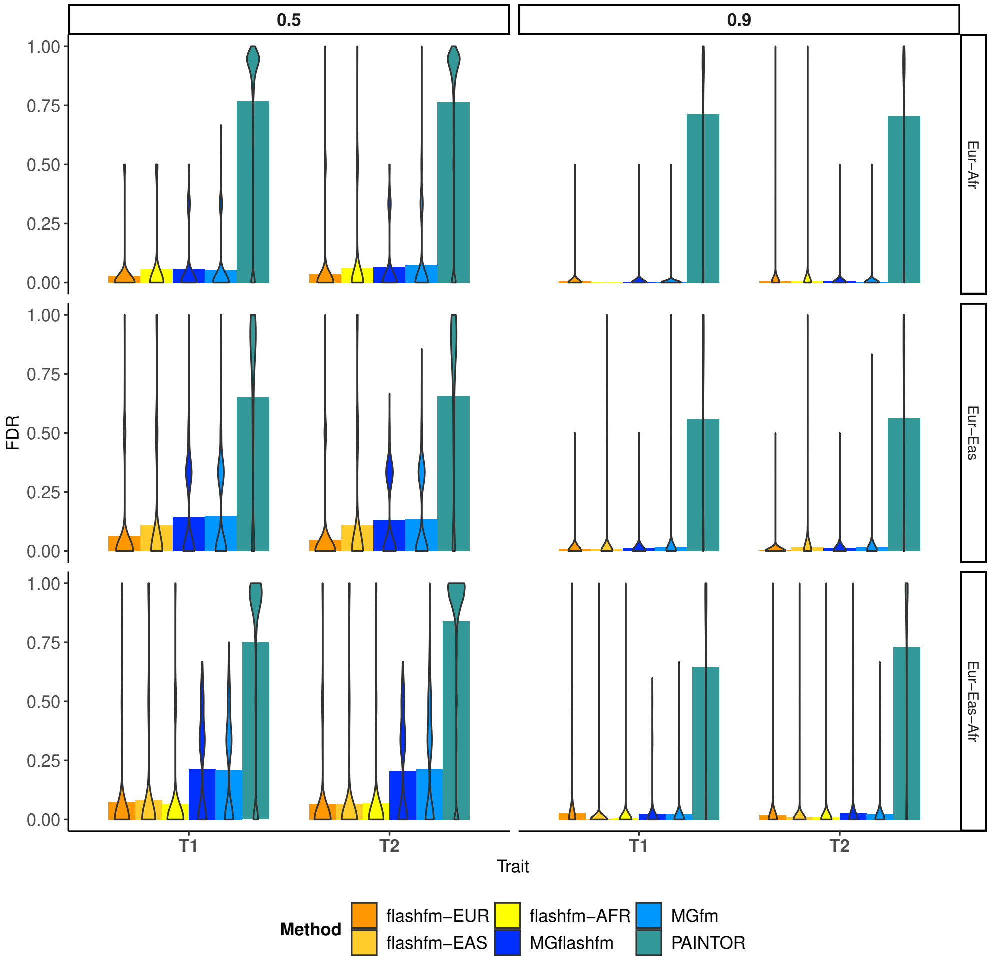

Supplementary Figure 4: **MGflashfm has the highest accuracy and resolution among calibrated methods for two traits in two groups.** For EUR-EAS simulations, three simulation settings are shown as either having equal sample sizes of 10k each or sample sizes of 90k EUR and 10k EAS, and either two causal variants for each trait with one shared (trait 1: AD, trait 2: AC) or non-overlapping causal variants and one trait having a single causal variant (trait 1: AD, trait 2: C); any pair of causal variants have $r^2 < 0.5$ and there are 300 replications within each setting. (a) Distribution of the minimum MPP of causal variants for each trait; the median is given by the centre line, upper and lower quartiles are the box limits, whiskers are at most 1.5x interquartile range, and width indicates the frequency. This indicates that MGflashfm is best at prioritising causal variants when the traits share a causal variant or similar performance to MGfm when no sharing. (b) Comparison of the sizes of 99% credible sets from MGflashfm and MGfm. This suggests that MGflashfm tends to have better resolution than MGfm.

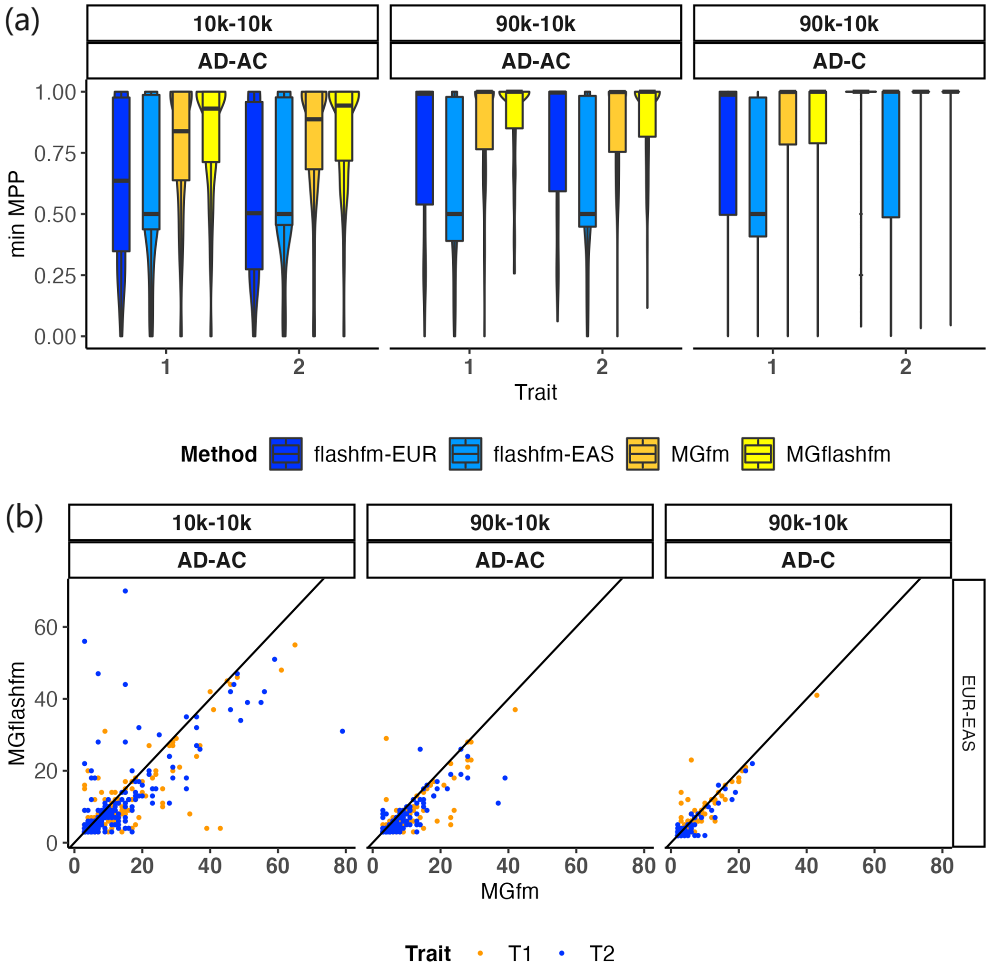

Supplementary Figure 5: **MGflashfm has the highest accuracy and resolution among calibrated methods for two traits in three groups.** For EUR-EAS-AFR simulations, two simulation settings are shown as either having equal sample sizes of 10k each or sample sizes of 90k EUR, 40k EAS, and 10k AFR. There are two causal variants for each trait with one shared (trait 1: AD, trait 2: AC) and any pair of causal variants have $r^2 < 0.5$; there are 300 replications within each setting. (a) Distribution of the minimum MPP of causal variants for each trait; the median is given by the centre line, upper and lower quartiles are the box limits, whiskers are at most 1.5x interquartile range, and width indicates the frequency. This indicates that MGflashfm is best at prioritising causal variants. (b) Comparison of the sizes of 99% credible sets from MGflashfm and MGfm. This suggests that MGflashfm tends to have better resolution than MGfm.

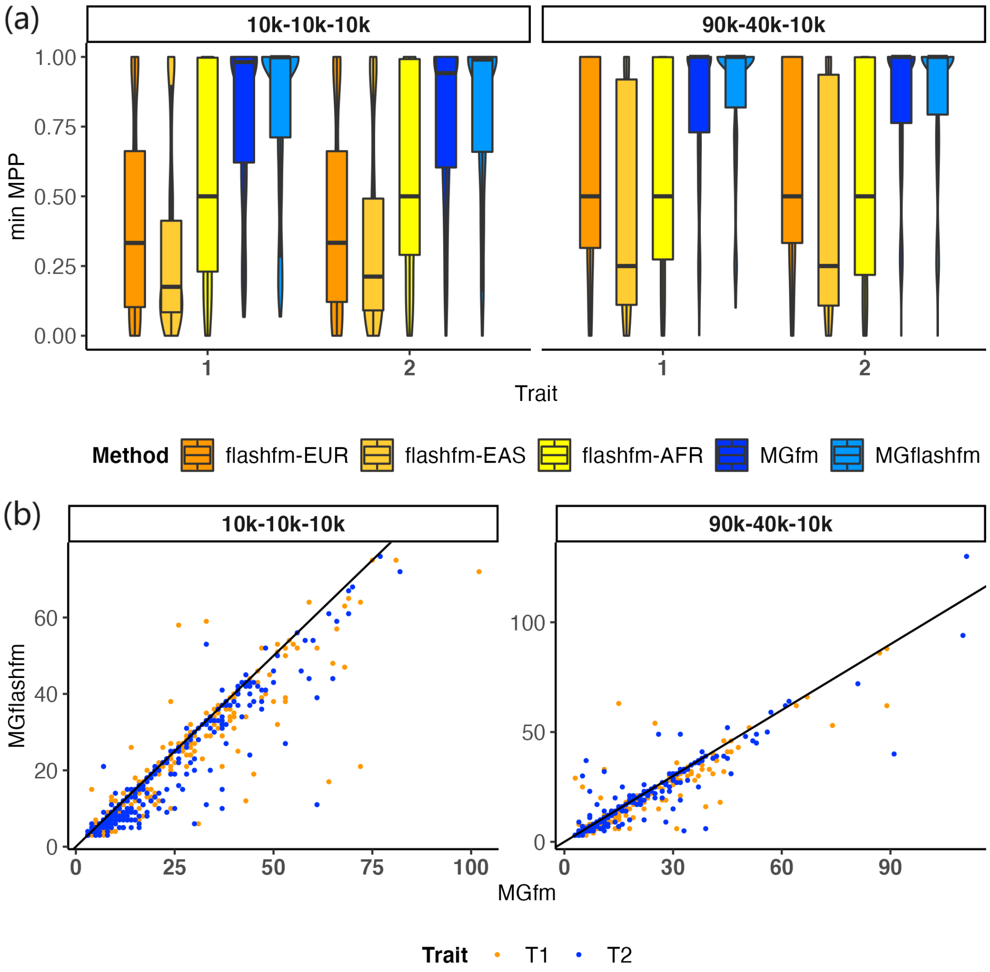

2 Supplementary Tables

Method	330-variant region	1610-variant region
**MGflashfm**	106.2<br>(91.9, 120.8)	684.3<br>(589.9, 717.3)
**MGfm**	59.8<br>(59.1, 58.0)	394.6<br>(380.6, 402.9)
**PAINTOR**	1664.4<br>(757.4, 3824.9)	1753.1<br>(1262.8, 2864.9)
**msCAVIAR**	1274.7<br>(1253.5, 1294.1)	>10 hours

Supplementary Table 1: **Computational time of multi-group methods with varying region size.** The median running times (with second and third quartiles) are given in seconds. Running time was measured over 100 replications in simulations of two traits in two groups. The traits had correlation 0.4 and sample sizes were 90,000 (EUR) and 10,000 (AFR). The APOE region chr19:45000000-45800000 (GRCh37/hg19), consisting of at most 1610 variants or a subset of at most 330 variants in a group, was used for all simulations.

Number of groups	2	3	4	5
**MGflashfm**	684.3<br>(589.9, 717.3)	761.7<br>(686.1, 842.7)	913.3<br>(831.9, 1015.3)	1089.5<br>(976.5, 1367.2)
**MGfm**	394.6<br>(380.6, 402.9)	452.9<br>(431.4, 467.5)	551.3<br>(533.7, 572.9)	622.7<br>(591.8, 890.0)

Supplementary Table 2: **Varying number of groups, median MGflashfm and MGfm running times (with second and third quartiles).** Median time was measured over 100 replications in simulations of 2 traits in 2-5 groups. Traits had correlation 0.4 and sample sizes ranged from 10,000 to 90,000 among groups. The APOE region chr19:45000000-45800000 (GRCh37/hg19), consisting of at most 1610 variants in a group, was used for all simulations.

Number of Traits	2	3	4
Computational Time	684.3	1024.9	1214.2
	(589.9, 717.3)	(920.1, 1190.3)	(1111.6, 1337.9)

Supplementary Table 3: **Computational time for MGflashfm with varying number of traits.** The median MGflashfm running time (with second and third quartiles) is given in seconds. Running time was measured over 300 replications in simulations of 2, 3, and 4 traits in two groups. The traits had correlation 0.4 and sample sizes were 90,000 (EUR) and 10,000 (AFR). The APOE region chr19:45000000-45800000 (GRCh37/hg19), consisting of at most 1610 variants in a group, was used for all simulations. As MGfm is for single traits, its speed is not considered here.

Method	T1: Pr(cvs in $\mathbf{CS99 \geq 1}$)	T1: Pr(cvs in $\mathbf{CS99 = 2}$)	T2: Pr(cv in $\mathbf{CS99 \geq 1}$)	T2: Pr(cvs in $\mathbf{CS99 = 2}$)
**MGflashfm**	1.0	0.960	1.0	0.970
		(0.933, 0.987)		(0.946, 0.994)
**MGfm**	1.0	0.955	1.0	0.960
		(0.926, 0.984)		(0.933, 0.987)
**flashfm-AFR**	1.0	0.965	1.0	0.970
		(0.940, 0.990)		(0.946, 0.994)
**flashfm-EUR**	0.995	0	1.0	0
	(0.985,1.0)			

Supplementary Table 4: **Calibration is retained by MGflashfm and MGfm upon exclusion of a causal variant in a group.** Two traits, each with two causal variants, were simulated in two groups. The causal variants for trait 1 are labeled as A, D and those for trait 2 are A, C, to indicate that one causal variant is shared between the traits. The A variant has MAF<0.01 in EUR and MAF>0.01 in AFR, and this is the variant that is removed from the EUR group. There are 200 replications.

3 Supplementary Methods

The MGflashfm framework builds on that of flashfm[1] for multi-trait fine-mapping, which leverages information between traits to improve precision when there are shared causal variant(s) between traits. Multi-group fine-mapping has potential to further improve precision of fine-mapping due to differences in linkage disequilibrium (LD) between diverse population groups. For simplicity, we first consider the context of single-trait fine-mapping, then extend to multi-group multi-trait fine-mapping.

We first focus on derivations for the two group setting, which easily extends to more than two groups. Assume that for a single quantitative trait, we have $N_j,\ j=1,2$ measurements of the trait within group j. Also, within each group, the traits are transformed to meet conditional normality and homogeneity assumptions, conditional on covariates. Later, as in flashfm, we relax this so that a subset of individuals may have missing measurements for some of the traits. Here, we find an expression for the multi-group ABF of causal variant models and show that the information from single group analyses could be used to evaluate the multi-group ABF.

To find expressions of the log(ABF) for each of the joint and marginal models we use the approximation based on the Bayesian information criterion (BIC) from the null and causal models (BIC_0 and BIC_1, respectively)[2]. The log(ABF) approximation $(\mathrm{BIC}_0 - \mathrm{BIC}_1)/2$, is expressed in terms of log likelihoods as

$$\log(\mathsf{ABF}) \doteq l_1 - l_0 - m\log(N)/2, \tag{1}$$

where m is the number of causal variants in the model and l_1 and l_0 are the log likelihoods of the causal and null models, evaluated at the maximum likelihood estimates. In flashfm[1], we show that the log(ABF) for a single trait j model of m_j variants, denoted by M_j, is

$$\begin{aligned}\log(\mathsf{ABF}_j) &= -\frac{N}{2}\log\left(\frac{(\mathbf{y}_j - \mathbf{X}_{M_j}\hat{\boldsymbol{\beta}}_j)^T(\mathbf{y}_j - \mathbf{X}_{M_j}\hat{\boldsymbol{\beta}}_j)}{\mathbf{y}_j^T\mathbf{y}_j}\right) - \frac{k_j}{2}\log(N) \\ &= -\frac{N}{2}\log\left(\frac{\hat{V}_{M_j}}{\hat{V}_j}\right) - \frac{k_j}{2}\log(N), \end{aligned} \tag{2}$$

where V_j is the variance of trait j and $\hat{V}_{M_j}$ is the residual variance from model M_j. The flashfm[1]

multi-trait BF for M traits is then shown to be:

$$\log(\mathsf{ABF}^{\mathsf{M}}) = \sum_{j=1}^{M} \log(\mathsf{ABF}_j) + D_M,$$

where

$$D_M = -\frac{N}{2}\left(\log\begin{vmatrix} 1 & \frac{h_{12}}{g_2} & \cdots & \frac{h_{1M}}{g_M} \\ \frac{h_{21}}{g_1} & 1 & \cdots & \frac{h_{2M}}{g_M} \\ \vdots & \frac{h_{i2}}{g_2} & \ddots & \vdots \\ \frac{h_{M1}}{g_1} & \frac{h_{M2}}{g_2} & \cdots & 1 \end{vmatrix} - \log\begin{vmatrix} 1 & \frac{C_{12}}{V_2} & \cdots & \frac{C_{1M}}{V_M} \\ \frac{C_{21}}{V_1} & 1 & \cdots & \frac{C_{2M}}{V_M} \\ \vdots & \frac{C_{i2}}{V_2} & \ddots & \vdots \\ \frac{C_{M1}}{V_1} & \frac{C_{M2}}{V_2} & \cdots & 1 \end{vmatrix}\right). \tag{3}$$

Let $N = N_1 + N_2$, and denote a group 1 model of m_1 variants by M_1 and, likewise, M_2 is a m_2-variant model for group 2. Using (1) and the fact that the groups are independent, we find that the joint Bayes factor for models M_1 and M_2 for groups 1 and 2, having m_1 and m_2 variants, respectively, is given by

$$BF^{(1,2)}_{M_1,M_2} = BF^{(1)}_{M_1} \times \left(\frac{N_1}{N}\right)^{\frac{m_1}{2}} \times BF^{(2)}_{M_2} \times \left(\frac{N_2}{N}\right)^{\frac{m_2}{2}} \tag{4}$$

A natural joint prior probability for a m_1-variant group 1 model with a m_2-variant group 2 model is $p^{(1,2)}_{m_1,m_2} = p_{m_1} p_{m_2}$, where p_{m_1} and p_{m_2} are prior probabilities of a m_1-variant group 1 model and m_2-variant group 2 model, respectively; this considers the full joint model search space. Assuming that the groups share at least one causal variant, we add the restriction that the joint prior is only non-zero when the models overlap.

In particular, denoting the size (number of variants) in a model $M^{(k)}$ for group k by $|M^{(k)}|$, we have

$$\begin{aligned}&\Pr(|M^{(1)}| = m_1, |M^{(2)}| = m_2)\\ &= \Pr(|M^{(1)}| = m_1, |M^{(2)}| = m_2, M^{(1)} \cap M^{(2)} \neq \varnothing) + \Pr(|M^{(1)}| = m_1, |M^{(2)}| = m_2, M^{(1)} \cap M^{(2)} = \varnothing).\end{aligned}$$

However, we set the prior to 0 when $M^{(1)} \cap M^{(2)} = \varnothing$, so we introduce a correction factor τ_{m_1,m_2} such that

$$\Pr(|M^{(1)}| = m_1, |M^{(2)}| = m_2) = \Pr(|M^{(1)}| = m_1, |M^{(2)}| = m_2, M^{(1)} \cap M^{(2)} \neq \varnothing)\tau_{m_1,m_2},$$

so that the total joint prior probability of a m_1-variant group 1 model with a m_2-variant group 2 model in the reduced search space is anchored to remain the same as in the full model search space.

Let $S = \{(i,j) : |M_i^{(1)}| = m_1, |M_j^{(2)}| = m_2\}$ and n be the number of SNPs in the region, then, we find τ_{m_1,m_2} as follows

$$\begin{aligned}\sum_{(i,j)\in S} p_{m_1} p_{m_2} &= \sum_{(i,j)\in S} p_{m_1} p_{m_2} 1\{M_i^{(1)} \cap M_j^{(2)} \neq \varnothing\} \tau_{m_1,m_2} \\ \binom{n}{m_1}\binom{n}{m_2} p_{m_1} p_{m_2} &= \left[\binom{n}{m_1}\binom{n}{m_2} - \binom{n}{m_1}\binom{n-m_1}{m_2}\right] p_{m_1} p_{m_2} \tau_{m_1,m_2},\end{aligned}$$

so that

$$\tau_{m_1,m_2} = \frac{\binom{n}{m_2}}{\binom{n}{m_2} - \binom{n-m_1}{m_2}} \tag{5}$$

So the joint prior probability is

$$p^{(1,2)}_{m_1,m_2} = p_{m_1} p_{m_2} 1\{M^{(1)} \cap M^{(2)} \neq \varnothing\} \tau_{m_1,m_2}, \tag{6}$$

where τ_{m_1,m_2} is the correction factor (5)

It follows from (4) and (6) that the joint posterior probability $PP^{(1,2)}_{M_1,M_2}$ for a particular model configuration $\{M_1, M_2\}$ may be found from only the $PP^{(1)}_{M_1}$, $PP^{(2)}_{M_2}$ of each model within their respective single-group fine-mapping model PPs, as follows:

$$\begin{aligned}PP^{(1,2)}_{M_1,M_2} &= p_{m_1} p_{m_2} BF^{(1)}_{M_1} \left(\frac{N_1}{N}\right)^{\frac{m_1}{2}} BF^{(2)}_{M_2} \left(\frac{N_2}{N}\right)^{\frac{m_2}{2}} 1\{M^{(1)} \cap M^{(2)} \neq \varnothing\} \tau_{m_1,m_2} \\ &= PP^{(1)}_{M_1} \left(\frac{N_1}{N}\right)^{\frac{m_1}{2}} PP^{(2)}_{M_2} \left(\frac{N_2}{N}\right)^{\frac{m_2}{2}} 1\{M^{(1)} \cap M^{(2)} \neq \varnothing\} \tau_{m_1,m_2} \end{aligned} \tag{7}$$

Let C be a set of variants that compose a a multi-group model. This encompasses all group 1 - group 2 models that share at least one variant and C is the collection of all variants in these models. So, the

multi-group PP for set C is given by

$$PP_C = \sum_{\substack{ij:M_i^{(1)} \cup M_j^{(2)} = C, \\ M_i^{(1)} \cap M_j^{(2)} \neq \varnothing}} PP_{ij}^{(1,2)} \tag{8}$$

Finally, for variant s, multi-group MPPs (marginal posterior probability - probability that the variant appears in a model) are found from

$$MPP_s = \sum_{c:s \in c} PP_C.$$

This framework allows us to first evaluate evidence for multi-variant models within each group, accounting for group-specific LD. Then, evaluate joint evidence for a particular model configuration $\{M_i, M_j\}$, of model M_i for group 1 with model M_j for group 2, having already accounted for LD within each group.

Now, assume that we have measurements of M quantitative traits within each group. We consider the same framework as above, but instead of making use of the single-trait fine-mapping model PPs from each group, we consider the multi-trait fine-mapping model PPs from flashfm applied to multiple traits within each group/study. This multi-group multi-trait fine-mapping approach is summarised by the following steps:

1. For each group, consider only variants that are present for all traits in that group, but do not intersect variants over groups;

2. Single-trait fine-mapping of each trait, within each group to obtain model PPs for each trait in each group;

3. Multi-trait fine-mapping (flashfm) within each group to leverage information between traits and obtain trait-adjusted model PPs for each trait in each group;

4. For each trait, use the above-described framework with $PP_{M_1}^{(1)}$ and $PP_{M_2}^{(2)}$ as found from multi-trait fine-mapping in groups 1 and 2, respectively.

This framework is extended to 3 groups, using similar arguments to the detailed 2-group setting previously described. Let $N = N_1 + N_2 + N_3$ and we have

$$BF^{(1,2,3)}_{M_1,M_2,M_3} = BF^{(1)}_{M_1} \times \left(\frac{N_1}{N}\right)^{\frac{m_1}{2}} \times BF^{(2)}_{M_2} \times \left(\frac{N_2}{N}\right)^{\frac{m_2}{2}} \times BF^{(3)}_{M_3} \times \left(\frac{N_3}{N}\right)^{\frac{m_3}{2}} \tag{9}$$

We assume that there is a shared causal variant between at least two of the three group models, which leads to a correction factor of

$$\tau_{m_1,m_2,m_3} = \frac{\binom{n}{m_2}\binom{n}{m_3}}{\binom{n}{m_2}\binom{n}{m_3} - \binom{n-m_1}{m_2}\binom{n-m_1-m_2}{m_3}};\ m_1 \geq m_2 \geq m_3 \geq 0. \tag{10}$$

So that the multi-group PP for a particular model configuration is

$$PP^{(1,2,3)}_{M_1,M_2,M_3} = PP^{(1)}_{M_1}\left(\frac{N_1}{N}\right)^{\frac{m_1}{2}} PP^{(2)}_{M_2}\left(\frac{N_2}{N}\right)^{\frac{m_2}{2}} PP^{(3)}_{M_3}\left(\frac{N_3}{N}\right)^{\frac{m_3}{2}} 1\{M_1 \cap M_2 \neq \varnothing \text{ or } M_1 \cap M_3 \neq \varnothing \text{ or } M_2 \cap M_3 \neq \varnothing\}\tau_{m_1,m_2,m_3}$$

and the multi-group PP for a set C of variants is given by

$$PP_C = \sum_{\substack{h,i,j: M^{(1)}_h \cup M^{(2)}_i \cup M^{(3)}_j = C, \\ (h,i,j) \in S}} PP^{(1,2,3)}_{h,i,j}, \tag{11}$$

where $S = \{(h,i,j) : M^{(1)}_h \cap M^{(2)}_i \neq \varnothing \text{ or } M^{(1)}_h \cap M^{(3)}_j \neq \varnothing \text{ or } M^{(2)}_i \cap M^{(3)}_j \neq \varnothing\}$

Supplementary References

[1] Hernandez N et al. "The flashfm approach for fine-mapping multiple quantitative traits". In: *Nat Comm* 12 (2021), p. 6147. DOI: 10.1038/s41467-021-26364-y.

[2] Wagenmakers E.-J. "A practical solution to the pervasive problems of p values". In: *Psychon. Bull. Rev.* 14 (2007), pp. 779–804. DOI: 10.3758/BF03194105.
