# Supplementary material for: Leveraging information between multiple population groups and traits improves fine-mapping resolution
Source: Nat Commun. 2023 Nov 10;14:7279. doi: 10.1038/s41467-023-43159-5 (PMC10638399; doi:10.1038/s41467-023-43159-5)
Supplement: Supplementary file 3 — Description of Additional Supplementary Files [file 41467_2023_43159_MOESM3_ESM.pdf]

# Description of Additional Supplementary Files

File Name: Supplementary Data 1
Description: Detailed results and summaries from the GLGC fine-mapping of four lipids traits across five population groups.
