# Supplementary material for: Leveraging information between multiple population groups and traits improves fine-mapping resolution
Source: Nat Commun. 2023 Nov 10;14:7279. doi: 10.1038/s41467-023-43159-5 (PMC10638399; doi:10.1038/s41467-023-43159-5)
Supplement: Supplementary file 5 — Reporting Summary [file 41467_2023_43159_MOESM5_ESM.pdf]

Corresponding author(s): Jennifer Asimit

Last updated by author(s): 23/10/23

Reporting Summary

Nature Portfolio wishes to improve the reproducibility of the work that we publish. This form provides structure for consistency and transparency in reporting. For further information on Nature Portfolio policies, see our Editorial Policies and the Editorial Policy Checklist.

Statistics

For all statistical analyses, confirm that the following items are present in the figure legend, table legend, main text, or Methods section.

n/a	Confirmed	
☐	☒	The exact sample size (*n*) for each experimental group/condition, given as a discrete number and unit of measurement
☒	☐	A statement on whether measurements were taken from distinct samples or whether the same sample was measured repeatedly
☐	☒	The statistical test(s) used AND whether they are one- or two-sided<br>*Only common tests should be described solely by name; describe more complex techniques in the Methods section.*
☐	☒	A description of all covariates tested
☐	☒	A description of any assumptions or corrections, such as tests of normality and adjustment for multiple comparisons
☐	☒	A full description of the statistical parameters including central tendency (e.g. means) or other basic estimates (e.g. regression coefficient) AND variation (e.g. standard deviation) or associated estimates of uncertainty (e.g. confidence intervals)
☐	☒	For null hypothesis testing, the test statistic (e.g. *F*, *t*, *r*) with confidence intervals, effect sizes, degrees of freedom and *P* value noted<br>*Give P values as exact values whenever suitable.*
☐	☒	For Bayesian analysis, information on the choice of priors and Markov chain Monte Carlo settings
☒	☐	For hierarchical and complex designs, identification of the appropriate level for tests and full reporting of outcomes
☐	☒	Estimates of effect sizes (e.g. Cohen's *d*, Pearson's *r*), indicating how they were calculated

Our web collection on statistics for biologists contains articles on many of the points above.

Software and code

Policy information about availability of computer code

Data collection	No software was used for data collection.
Data analysis	Our proposed multi-group fine-mapping methods are available at https://jennasimit.github.io/MGflashfm/ ((DOI: 10.5281/zenodo.797453533) and custom code for our analysis of the GLGC data is available at https://github.com/fz-cambridge/MGflashfm-GLGC-analysis (DOI: 10.5281/zenodo.10034536). Trait genetic correlations were estimated using LD scores (v1.0.1, https://github.com/bulik/ldsc) together with MTAR (http://www.github.com/baolinwu/MTAR). We simulated genotype data with hapgen2 (http://mathgen.stats.ox.ac.uk/genetics_software/hapgen/hapgen2.html). The annotation tool we used is Ensembl Variant Effect Predictor (VEP) GRCh37 (https://grch37.ensembl.org/info/docs/tools/vep/index.html).

For manuscripts utilizing custom algorithms or software that are central to the research but not yet described in published literature, software must be made available to editors and reviewers. We strongly encourage code deposition in a community repository (e.g. GitHub). See the Nature Portfolio guidelines for submitting code & software for further information.

Data

Policy information about availability of data

All manuscripts must include a data availability statement. This statement should provide the following information, where applicable:

- Accession codes, unique identifiers, or web links for publicly available datasets
- A description of any restrictions on data availability
- For clinical datasets or third party data, please ensure that the statement adheres to our policy

The GLGC lipids traits GWAS summary statistics from five genetically similar groups are freely available from http://csg.sph.umich.edu/willer/public/glgc-lipids2021/results/ancestry_specific/. Reference panels for LD and LD scores were generated from the 1000 Genomes data available at https://ctg.cncr.nl/software/MAGMA/ref_data/. The detailed data results of our multi-group multi-trait fine-mapping GLGC results are given in Supplementary Data 1. For ease of access they are also deposited in a FigShare public data repository (https://doi.org/10.6084/m9.figshare.2326670332). Positions are given according to hg19/build 37.

Research involving human participants, their data, or biological material

Policy information about studies with human participants or human data. See also policy information about sex, gender (identity/presentation), and sexual orientation and race, ethnicity and racism.

Reporting on sex and gender	We used the sex-combined GWAs summary statistics provided by the GLGC.
Reporting on race, ethnicity, or other socially relevant groupings	We used the population groupings previously defined by the GLGC, which are labelled by continent. These population groupings are based on genetic similarity to avoid problems of structure in the data that could lead to false-positives in genetic studies.
Population characteristics	As above.
Recruitment	We used previously collected data.
Ethics oversight	We used previously collected data.

Note that full information on the approval of the study protocol must also be provided in the manuscript.

Field-specific reporting

Please select the one below that is the best fit for your research. If you are not sure, read the appropriate sections before making your selection.

☒ Life sciences ☐ Behavioural & social sciences ☐ Ecological, evolutionary & environmental sciences

For a reference copy of the document with all sections, see nature.com/documents/nr-reporting-summary-flat.pdf

Life sciences study design

All studies must disclose on these points even when the disclosure is negative.

Sample size	We used samples that were previously collected by GLGC. As stated by GLGC, the sample sizes were deemed to be sufficient, as they are at least as large as GWAS studies of related quantitative traits that have successfully identified associated genetic variants.
Data exclusions	No data were excluded.
Replication	In our simulation studies we use 300 replications of each simulation setting and show that our proposed methods are well-calibrated with higher power than other methods and similarly low or lower false discovery rate (FDR). In our fine-mapping of the GLGC lipids traits, we identify likely causal variants and provide posterior probabilities of being causal. We compare our fine-mapping results with previously published results (same data, different methods) and show replication of GLGC prioritised variants in Supplementary Data 1.2, and also identify variants that have not been previously prioritised by any of the GLGC analyses (MGflashfm allows multiple causal variants, whilst the GLGC analyses used methods that assume a single causal variant). We prioritise 19 variants that are missense, stop-gained or splice variants; 63% (12/19) of these variants are not identified by any of the GLGC fine-mapping analyses (Supplementary Data 1.3). Among all traits, MGflashfm prioritised 185 unique variants, of which 77% are new compared to any of the GLGC analyses (EUR, AFR, multi-group); 168 variants are prioritised by MGfm (Supplementary Data 1.4).
Randomization	This is not relevant to our study as individuals were not assigned to groups.
Blinding	Blinding was not relevant to our study as individuals were not assigned to groups.

Reporting for specific materials, systems and methods

We require information from authors about some types of materials, experimental systems and methods used in many studies. Here, indicate whether each material, system or method listed is relevant to your study. If you are not sure if a list item applies to your research, read the appropriate section before selecting a response.

Materials & experimental systems

n/a	Involved in the study
☒	☐ Antibodies
☒	☐ Eukaryotic cell lines
☒	☐ Palaeontology and archaeology
☒	☐ Animals and other organisms
☒	☐ Clinical data
☒	☐ Dual use research of concern
☒	☐ Plants

Methods

n/a	Involved in the study
☒	☐ ChIP-seq
☒	☐ Flow cytometry
☒	☐ MRI-based neuroimaging
